# Differences exist across insurance schemes in China post-consolidation

**DOI:** 10.1371/journal.pone.0187100

**Published:** 2017-11-10

**Authors:** Yang Li, Yinjun Zhao, Danhui Yi, Xiaojun Wang, Yan Jiang, Yu Wang, Xinchun Liu, Shuangge Ma

**Affiliations:** 1 School of Statistics, Renmin University of China, Beijing, China; 2 Center for Applied Statistics, Renmin University of China, Beijing, China; 3 Statistical Consulting Center, Renmin University of China, Beijing, China; 4 School of Public Health, Yale University, New Haven, CT, United States of America; Old Dominion University, UNITED STATES

## Abstract

**Background:**

In China, the basic insurance system consists of three schemes: the UEBMI (Urban Employee Basic Medical Insurance), URBMI (Urban Resident Basic Medical Insurance), and NCMS (New Cooperative Medical Scheme), across which significant differences have been observed. Since 2009, the central government has been experimenting with consolidating these schemes in selected areas. This study examines whether differences still exist across schemes after the consolidation.

**Methods:**

A survey was conducted in the city of Suzhou, collecting data on subjects 45 years old and above with at least one inpatient or outpatient treatment during a period of twelve months. Analysis on 583 subjects was performed comparing subjects’ characteristics across insurance schemes. A resampling-based method was applied to compute the predicted gross medical cost, OOP (out-of-pocket) cost, and insurance reimbursement rate.

**Results:**

Subjects under different insurance schemes differ in multiple aspects. For inpatient treatments, subjects under the URBMI have the highest observed and predicted gross and OOP costs, while those under the UEBMI have the lowest. For outpatient treatments, subjects under the UEBMI and URBMI have comparable costs, while those under the NCMS have much lower costs. Subjects under the NCMS also have a much lower reimbursement rate.

**Conclusions:**

Differences still exist across schemes in medical costs and insurance reimbursement rate post-consolidation. Further investigations are needed to identify the causes, and interventions are needed to eliminate such differences.

## Background

China has one of the largest health insurance systems in the world. In China, commercial health insurance is underdeveloped, and the government-funded basic health insurance dominates. It is estimated that by 2010, at least 95% of the population was covered by the basic health insurance [1]. The basic insurance system consists of three schemes [2]. Specifically, the NCMS (New Cooperative Medical Scheme) covers residents in rural areas (which are defined by “HuKou”–the official residency registration). The UEBMI (Urban Employee Basic Medical Insurance) scheme covers urban residents that are employed. The URBMI (Urban Resident Basic Medical Insurance) scheme covers urban residents not covered by the UEBMI, including the unemployed, seniors, and children. The three schemes are governed by different agencies. NCMS is governed by the Chinese National Health and Family Planning, whereas URBMI and UEBMI are governed by the Chinese Ministry of Human Resources and Social Security. The major funding source is government subsidies for NCMS and URBMI and payroll taxes for UEBMI. The pool levels for the three schemes are also different in that NCMS funds are pooled at the county level whereas URBMI and UEBMI funds are pooled at the municipal level. The aforementioned differences have led to differences in regulations, coverage depths, reimbursement processes, and other aspects within and across schemes [3]. Published studies [4–6] have conducted across-scheme comparisons and reported significant differences in health care, financial consequences of illness, and health outcomes.

Across-region/area variations in health care, insurance, and outcomes in China have attracted extensive attention. For example, one study examined the changes in the structure of total health expenditures between 2000 and 2011 and investigated the financial burdens of health care, with a particular emphasis on the differences between rural and urban areas (which were covered by different insurance schemes) and across regions with different economic statuses [7]. Using national survey data collected between 2003 and 2008, Jian and others analyzed the changes in the rural-urban care gap, which was partly caused by the difference in insurance, for people with chronic diseases [8]. Our literature review suggests that the observed differences (variations) are usually at least partly associated with the difference in insurance schemes.

The Chinese central government set the goal of achieving universal health insurance coverage by 2020 [3]. To eliminate disparity and enhance efficiency, a major step is to consolidate the three basic insurance schemes. The consolidation includes merging administrative offices, integrating funding pools, unifying benefit levels and payment systems, and others. Detailed descriptions of the consolidation have been provided in the literature [3, 9–12] and will not be reiterated here. China is a huge country, and the consolidation process is very complex. As such, it is not realistic to carry out the consolidation throughout the whole country at the same time. Realizing this problem, the Chinese central government first conducted pilot experiments in selected areas. It was reported that seven provincial administrative regions (including the municipalities of Tianjin and Chongqing, Qinghai, Zhejiang, and Shandong provinces, Ningxia Hui autonomous region, and Xinjiang Production and Construction Corps) were in the process of consolidation by the middle of 2014 [3].

The consolidation is both time- and resource-consuming and has important implications and big impact. It is of significant interest to examine the consolidation process and, more importantly, its consequences. The philosophical, social, and managerial aspects of the consolidation have been examined at the macro level. Examples include the study by Sun and others, which analyzed the inequality in health care with a special emphasis on health insurance reimbursement and suggested the necessity of consolidation [13]. Wang and others conducted a survey study in four cities, analyzed people’s willingness toward consolidating the health insurance schemes, and suggested the importance of equal financing and equal benefit [14]. Published studies have suggested certain successes of the consolidation, for example, the positive association between the consolidation of NCMS and URBMI and health care accessibility and efficiency of the system. More specifically, a study in five cities showed that the reimbursement for inpatient treatment increased and the total expense decreased after the consolidation of NCMS and URBMI. An 8% increase in reimbursement was reported in areas with a consolidated scheme compared to those without consolidation. Our limited literature review suggests that all of the aforementioned and other published studies are based on hospital or government databases, which, although useful, are limited in lacking certain micro and personal information [3].

Complementary to the existing literature, the objective of this study is to examine the existence and extent of differences across the three insurance schemes after the consolidation. Such an investigation can provide important insights into the effectiveness of the consolidation and serve as the basis for future policy development and implementation. This study differs fundamentally from the existing ones in multiple aspects. Specifically, it focuses on cost and reimbursement and takes a perspective different from those philosophical, social, and managerial studies. Data were collected using a survey of random samples, which can effectively avoid the selection bias problem of hospital- and community-based studies. In addition, with the survey, we were able to collect more detailed information from a patients’ perspective. Last but not least, more comprehensive statistical analysis is conducted, which can better describe the net difference across schemes. This study can therefore be useful to public health researchers and policymakers in China as well as countries/regions with similar health insurance systems.

## Methods

### Data collection

This study was approved by a research ethics review committee at the Renmin University of China. Each participant was asked to sign an informed consent form. The survey was conducted in August of 2014 in the city of Suzhou, which is located in the Jiangsu province and economically highly developed. In 2014, Suzhou had a per capita GDP (gross domestic product) of 19,571 USD, compared to 7,138 USD for the whole China [15]. In Suzhou, the consolidation started in April of 2008, when rural residents (as defined by “Hukou”) employed off-farm, no matter in rural or urban areas, began to be insured by the UEBMI. The consolidation of the NCMS and URBMI also started with the merger of administrative offices, integration of funding pools, unification of benefit packages, and others [16, 17]. By the end of 2011, the three systems were administratively mostly consolidated [18].

A two-stage sampling was conducted. In the first stage, eight communities/villages were randomly selected, taking into account the rural/urban population ratio and economic status. In the second stage, samples were randomly selected within each community/village. The target sample size was 640, which was determined by resource availability. For each interviewed subject, information was first collected to determine eligibility. An interviewee was excluded if he/she was younger than 45 years old, did not receive any inpatient/outpatient treatment during a period of twelve months prior to the survey, or was not able to provide reliable information on treatments. The middle-aged and elderly were selected because they experienced health issues more frequently and had a greater demand for health care and insurance services. As the focus of this study is on medical cost and insurance, those who did not receive any treatment were excluded. A total of 668 subjects finished the survey, with a response rate of 62%. We were able to collect basic information on those who rejected. Analysis did not suggest any significant selection bias. Further examination of data suggested additional invalid records. Final analysis was conducted on 583 subjects with valid records. For each subject, information is available on demographics and inpatient and outpatient treatments. The average annual income of the subjects is 40, 112 RMB, comparable to that of the Suzhou population (39, 780RMB in 2014 [15]).

### Data analysis

A careful exploratory analysis was first conducted, and no obvious outlier was identified. With their significant differences, inpatient and outpatient treatments were separately analyzed.

In the first set of analysis, subjects’ characteristics were compared across the three insurance schemes using Chi-squared test and Fisher’s exact test for categorical variables and ANOVA for continuous variables. As in published studies [19], insurance utilization status was taken into consideration in the comparison. Under the current basic insurance system in China, insurance coverage does not indicate automatic insurance utilization. That is, an insured subject may or may not use insurance for a specific treatment episode. In principle, a subject can have different insurance utilization statuses for different treatment episodes. However, examination of data suggested that insurance utilization status is very consistent across episodes and hence can be represented by a single variable in this study.

The second set of analysis covers gross medical cost, OOP (out-of-pocket) cost, and insurance reimbursement rate. The gross cost includes the cost of treatment, transportation, food, and accommodation (that is associated with illness), medicine and supplies outside of the hospital, unofficial gift (to doctors, nurses, escorts, and caretakers), and lost income. The OOP cost is the gross cost subtracting the insurance reimbursement, if insurance is used. The reimbursement rate is the ratio of insurance reimbursement over gross cost.

Differences in medical costs and insurance reimbursement can be caused by differences in insurance schemes as well as differences in subjects’ characteristics. For example, published studies have suggested that education is directly associated with healthcare pursuit behaviors and medical cost. Different insurance schemes cover different populations, and so differences in personal characteristics inevitably exist across schemes. It is of more interest to quantify the net differences in medical costs and reimbursement caused by differences in insurance schemes while properly eliminating the effects of personal characteristics. As suggested by extensive causal inference studies, multivariate regression, which adjusts for confounders, cannot fully solve the problem. To this end, the following analysis strategy is implemented. Although sharing a similar spirit with causal inference analysis, it differs in that the “standard” causal inference analysis only generates an estimate of variable (insurance scheme in this case) effect, while the proposed analysis generates the *predicted values* of response variables (costs and reimbursement rates).

The analysis consists of the following steps. Denote *X* as the vector of potentially relevant factors (covariates) and *Y* as the response variable.

a) For the gross and OOP costs, consider the linear regression model *Y* = *α* + *β*′*X* + *ϵ*, where *α* is the intercept, *β* is the vector of regression coefficients, and *ε* is the random error. For the reimbursement rates (which lie between 0 and 1), consider the logistic-type regression, where *E*(*Y*) = exp(*α* + *β*′*X*)/(1 + exp(*α* + *β*′*X*)), and *E*(.)denotes expectation.
b) For subjects under each insurance scheme separately, conduct robust model estimation. Medical cost data do not have a normal distribution (usually with a long right tail), and observations on the distribution of reimbursement rate data have been conflicting. Robust estimation (as opposed to the ordinary maximum likelihood estimation) is adopted to accommodate non-normal distributions. Use the subscript “*i*” to denote the *i*th (*i* = 1,…,*n*) subject. For example, for cost, the estimate is defined as
  $$(\hat{\alpha },\hat{\beta })=argmin\;\sum_{i=1}^{n}\left|Y_{i}-(\alpha +\beta^{\prime }X_{i})\right|.$$
  Here, median regression, which is a special case of quantile regression, is adopted. Another way of accommodating non-normal distributions is transformation. However, with transformations, models are not on the original scale, and hence interpretations can be difficult.
c) Compute the predicted cost/reimbursement rate. Specifically, for each type of insurance scheme,
(c.1) randomly sample *n* subjects from the whole cohort with replacement. This sampling step ensures that different insurance schemes have the same distribution of subjects’ characteristics;
(c.2) for a subject with covariate value $\tilde{X}$, compute the predicted cost as
  $$\tilde{Y}=\hat{\alpha }+\hat{\beta }\prime \tilde{X}$$
  and predicted reimbursement rate as
  $$\tilde{Y}=\exp\left(\hat{\alpha }+\hat{\beta }\prime \tilde{X}\right)/(1+\exp\left(\hat{\alpha }+\hat{\beta }\prime \tilde{X}\right));$$
(c.3) to avoid bias caused by an extreme sampling, repeat steps (c.1) and (c.2) 1,000 times;
(c.4) compute summary statistics using the results generated in (c.3). As robust regression is adopted, median and MAD (median absolute deviation) are used for summarizing.

This analysis is built on sound statistical principles and popular techniques and can be easily realized.

Variables included in analysis represent subjects’ demographics, socioeconomic status, and healthcare quality and accessibility. They are age, sex, marital status, education, income, hospital visit frequency (specifically, admission days for inpatient treatment and visit number for outpatient treatment), healthcare grade (specifically, percentage of admission days in grade II and III hospitals for inpatient treatment and grade of the nearest hospital for outpatient treatment), and healthcare accessibility (specifically, weighted distance to admitted hospitals for inpatient treatment and distance to the nearest hospital for outpatient treatment). For the analysis reimbursement rates, costs are also included. These variables have been selected based on published literature because of their health economic implications. As our goal is to accurately predict costs and reimbursement rates (as opposed to identifying significant contributing factors), all of the aforementioned variables, statistically significant or not, are included in analysis. Analysis with selected significant variables has also been explored and led to similar results (details omitted).

## Results

### Characteristics of the whole cohort

**Fig 1:**
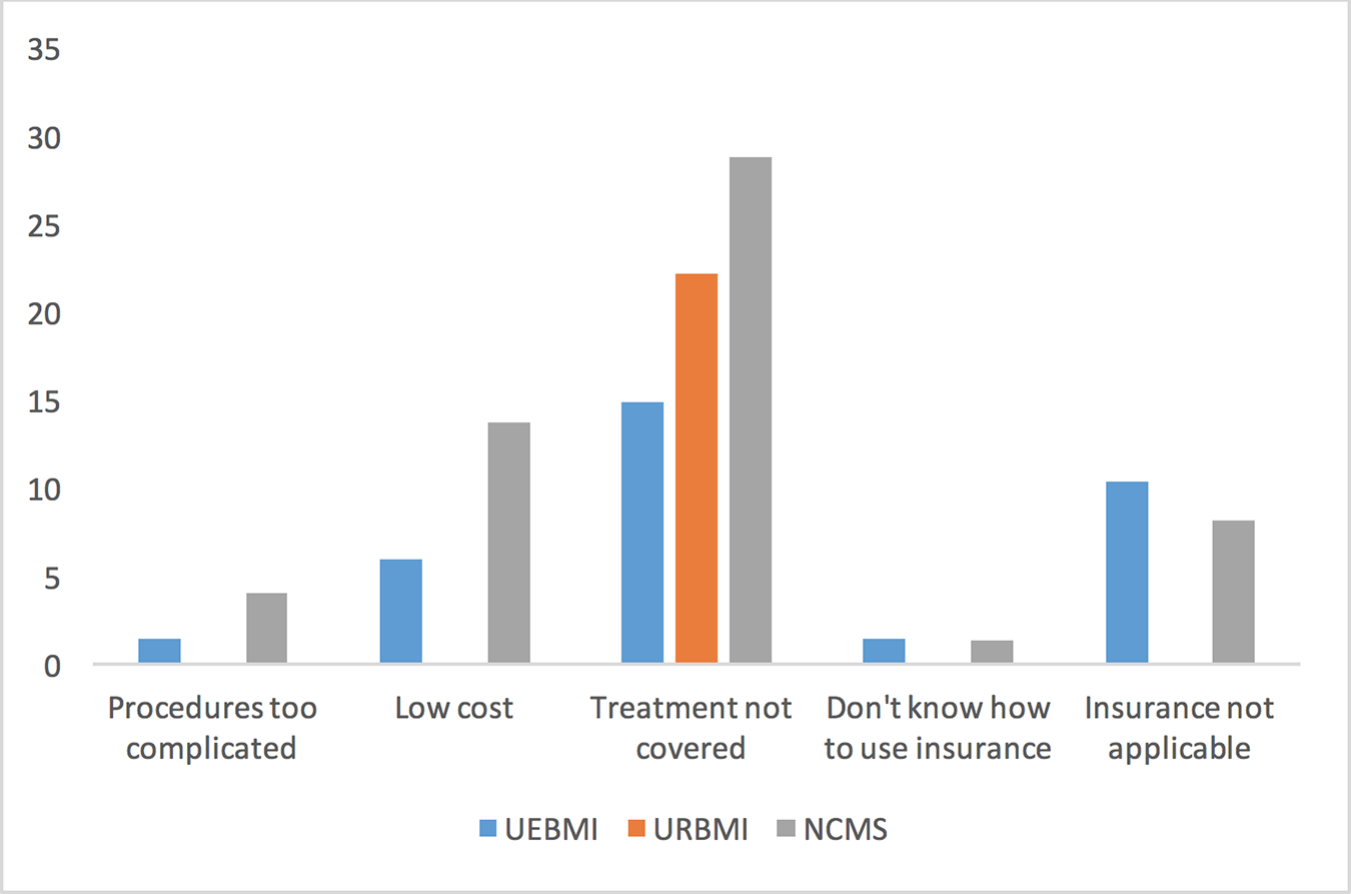
Reasons for not using insurance for outpatient treatment.

Among the surveyed subjects, 49.9% are male. The average age is 67.3 years old (sd = 9.9 years). During a period of twelve months, 262 subjects received inpatient treatments, and 543 received outpatient treatments. A total of 94.8% of the subjects were covered by basic insurance, with 55.4%, 16.0%, and 23.4% covered by the UEBMI, URBMI, and NCMS schemes, respectively. Insurance was used for all inpatient treatments. For outpatient treatments, the overall insurance utilization rate is 71%. Reasons for not using insurance are summarized in Fig 1. The most notable reason is that a specific treatment was not covered by insurance, followed by insurance not being applicable and the low cost of a treatment.

### Differences in subjects’ characteristics across insurance schemes

For inpatient treatments, the results are presented in Table 1. Subjects under different schemes are found to differ in multiple aspects. Specifically, a difference is observed in education (p-value < 0.001), with those under URBMI and NCMS having higher rates of primary school completion or being less educated. Occupation also differs significantly across schemes (p-value < 0.001). Another factor found to differ is income, with those under UEBMI having the highest income (mean 29.7k RMB, compared to 22.6k and 16.8k under URBMI and NCMS, respectively). A significant difference is observed in the utilization of health care facility. In China, the dominating majority of hospitals are public and under a strict grading system, with grade III hospitals offering the best quality of care (and being the most expensive). For each subject, the percentages of hospitalization days in grade III and other hospitals were computed. Table 1 shows that, under UEBMI, the majority of hospitalizations were in grade III hospitals, while the percentage is much lower under NCMS. The observed reimbursement rate also differs significantly (p-value < 0.001) and equals 72.8% (UEBMI), 71.5% (URBMI), and 55.6% (NCMS), respectively.

**Table 1:** Characteristics of subjects with inpatient treatments.

	UEBMI	URBMI	NCMS	p-value
	n = 137	n = 52	n = 73	
**Sex**				0.453
Male	73(53.3)	24(46.2)	42(57.5)	
Female	64(46.7)	28(53.8)	31(42.5)	
**Marital status**				0.877
Single/Divorced/Widowed	25(18.2)	11(21.2)	13(17.8)	
Married	112(81.8)	41(78.8)	60(82.2)	
**Education**				< .0001
Primary and less	43(31.4)	27(51.9)	49(67.1)	
Junior school	52(38)	12(23.1)	19(26)	
Senior school and more	42(30.7)	13(25)	5(6.8)	
**Occupation**				<0.001
Government	4(2.9)	—	—	
Enterprises	6(4.4)	3(5.8)	1(1.4)	
Farmers	1(0.7)	4(7.7)	28(38.4)	
Private Business	4(2.9)	5(9.6)	4(5.5)	
Retired	116(84.7)	18(34.6)	13(17.8)	
Unemployed	2(1.5)	14(26.9)	21(28.8)	
Others	4(2.9)	8(15.4)	6(8.2)	
**Weighted distance to hospital of treatment (meter)**				0.09
≤1000	76(55.5)	30(57.7)	30(41.1)	
>1000	61(44.5)	22(42.3)	43(58.9)	
**Age**	69.2(9.7)	66.5(9.8)	66.9(9.9)	0.141
**Income (1k)**	29.7(16.8)	22.6(9.9)	16.8(13.7)	< .0001
**Days of treatment**	22.9(31)	17.4(18.9)	20.9(41)	0.569
**Percentage of days in grade III hospital(s)**	0.88(0.32)	0.75(0.43)	0.47(0.5)	< .0001
**Percent of days in grade II hospital(s)**	0.1(0.29)	0.15(0.36)	0.27(0.44)	0.005
**Treatment cost (1k)**	22(28)	20.5(39.1)	18(35.4)	0.706
**Gross cost (1k)**	24.2(31.4)	22.4(39.7)	19.8(37)	0.689
**OOP cost (1k)**	8.5(14.8)	7.2(9.2)	10.4(27.8)	0.614
**Reimbursement rate**	0.73(0.15)	0.71(0.17)	0.56(0.18)	< .0001

For categorical variables, counts (percentage); for continuous variables, mean (sd).

For outpatient treatments, the results are presented in Table 2. Seventy-nine percent of the subjects under UEBMI used insurance, compared to 77% under URBMI and 47% under NCMS. In published studies, subjects who had but did not use insurance were found to have characteristics significantly different from those who used insurance [19]. Thus, as shown in Table 2, a comparison was conducted separately for different insurance utilization statuses. In both insurance utilization groups, education differs significantly across schemes (p-value < 0.001), with those under UEBMI being more highly educated. A difference in education is also observed between the two utilization groups. For example, under UEBMI, in the group that did not use insurance, 40.3% had completed primary school or were less educated, compared to 25.3% in the group that used insurance. In both utilization groups, occupation differs significantly across schemes (p-value < 0.001). Following published studies [19, 20], information was collected on the type of the nearest hospital, which serves as a surrogate for health care accessibility. A significant difference was observed across schemes. Under UEBMI, 50.7% (insurance not used) and 47.9% (insurance used) had the nearest hospitals being grade III, compared to 9.5% (insurance not used) and 9% (insurance used) under NCMS. Income also differs significantly across schemes (p-value < 0.001), with those under UEBMI having the highest income and those under NCMS having the lowest. In the group that used insurance, the number of treatments and treatment cost also differ significantly across schemes (p-values = 0.011 and 0.004, respectively). Those under UEBMI received the most treatments and had the highest treatment cost. Accordingly, they also have the highest gross medical cost (5.6k, compared to 3.9k under URBMI and 2.9k under NCMS, p-value = 0.033). When insurance was used, those under UEBMI and URBMI had a significantly higher reimbursement rate.

**Table 2:** Characteristics of subjects with outpatient treatments.

Insurance Utilization	No (n = 158)				Yes (n = 385)			
	UEBMI	URBMI	NCMS	p-value	UEBMI	URBMI	NCMS	p-value
	n = 67	n = 18	n = 73		n = 257	n = 61	n = 67	
**Sex**				0.772				0.353
Male	35(52.2)	11(61.1)	41(56.2)		122(47.5)	25(41)	36(53.7)	
Female	32(47.8)	7(38.9)	32(43.8)		135(52.5)	36(59)	31(46.3)	
**Marital status**				0.608				0.967
Single/Divorced/Widowed	11(16.4)	3(16.7)	8(11)		49(19.1)	11(18)	12(17.9)	
Married	56(83.6)	15(83.3)	65(89)		208(80.9)	50(82)	55(82.1)	
**Education**				<0.001				<0.001
Primary and less	27(40.3)	6(33.3)	51(69.9)		65(25.3)	24(39.3)	44(65.7)	
Junior school	20(29.9)	8(44.4)	18(24.7)		104(40.5)	20(32.8)	20(29.9)	
Senior school and more	20(29.9)	4(22.2)	4(5.5)		88(34.2)	17(27.9)	3(4.5)	
**Occupation**				<0.001				<0.001
Government	4(6)	—	—		4(1.6)	—	1(1.5)	
Enterprises	3(4.5)	1(5.6)	1(1.4)		20(7.8)	2(3.3)	2(3)	
Farmers	2(3)	2(11.1)	34(46.6)		2(0.8)	3(4.9)	28(41.8)	
Private Business	1(1.5)	4(22.2)	4(5.5)		6(2.3)	2(3.3)	4(6)	
Retired	51(76.1)	5(27.8)	9(12.3)		214(83.3)	36(59)	9(13.4)	
Unemployed	2(3)	2(11.1)	20(27.4)		3(1.2)	12(19.7)	18(26.9)	
Others	4(6)	4(22.2)	5(6.8)		8(3.1)	6(9.8)	5(7.5)	
**Distance to the nearest hospital (meter)**				0.141				0.218
≤1000	24(35.8)	11(61.1)	28(38.4)		126(49)	27(44.3)	25(37.3)	
>1000	43(64.2)	7(38.9)	45(61.6)		131(51)	34(55.7)	42(62.7)	
**Type of the nearest hospital**				<0.001				<0.001
Grade I	23(34.3)	8(44.4)	56(76.7)		122(47.5)	36(59)	60(89.6)	
Grade II	10(14.9)	2(11.1)	10(13.7)		12(4.7)	9(14.8)	1(1.5)	
Grade III	34(50.7)	8(44.4)	7(9.6)		123(47.9)	16(26.2)	6(9)	
**Age**	66.8(10.4)	64.5(9.6)	65.9(10.3)	0.664	68.3(9.4)	66.9(9.5)	66.1(10.3)	0.198
**Income (1k)**	30.9(24.2)	19.5(7.6)	16(16)	<0.001	30.2(14.5)	25(12.9)	15.8(10.8)	<0.001
**Number of treatment**	8.8(8.1)	3(1.8)	8.2(19.6)	0.789	9.2(10.1)	6.9(6.8)	5.9(4.5)	0.011
**Treatment cost (1k)**	2.6(3)	0.3(0.2)	3.7(10)	0.646	4.4(4.8)	3.5(4.3)	2.3(3.7)	0.004
**Gross Cost (1k)**	2.9(3.1)	3.3(4.7)	4.1(10)	0.824	5.6(9.4)	3.9(4.7)	2.9(4.3)	0.033
OOP Cost (1k)^*^	—	—	—	—	2.8(8.3)	1.8(3)	1.8(3.8)	0.456
**Reimbursement rate**	—	—	—	—	0.71(0.2)	0.69(0.3)	0.54(0.3)	<0.001

^*^ When insurance is not used, the OOP cost is equal to the gross cost.

### Differences in predicted cost and reimbursement rate

**Fig 2:**
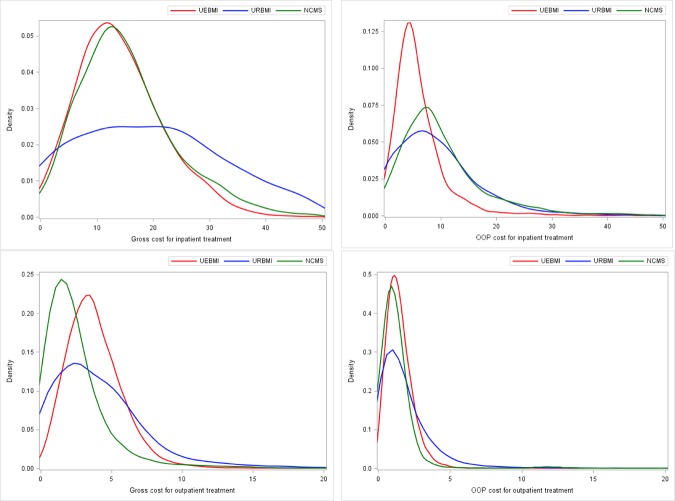
Density of predicted gross and OOP cost (1k RMB) for inpatient and outpatient treatments. Top left: Gross cost for inpatient treatment. Top right: OOP cost for inpatient treatment. Bottom left: Gross cost for outpatient treatment. Bottom right: OOP cost for outpatient treatment.

**Fig 3:**
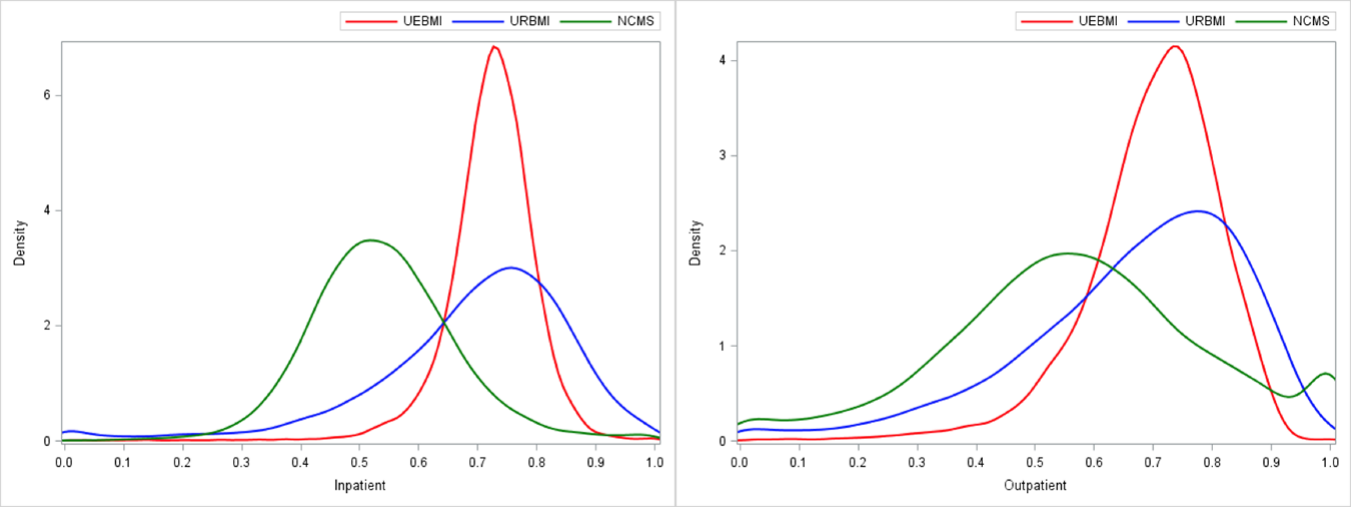
Density of predicted reimbursement rate. Left: Inpatient. Right: Outpatient.

With the analysis approach described above, distributions of the predicted gross and OOP costs and reimbursement rates can be generated. The distribution densities are plotted in Figs 2 and 3. The median and MAD, which are commonly used in robust analysis, are summarized in Table 3. For comparison, summaries of the observed values are also provided. For each comparison across the three schemes presented in Figs 2 and 3, the difference is significant with p-value < 0.001. Specifically, for inpatient treatments, the URBMI scheme has the highest predicted gross cost (median 19.9k), followed by the NCMS (15.07k) and then the UEBMI (14.26k). However, when factoring in insurance reimbursement, the NCMS scheme has the highest OOP cost (median 8.47k), while the UEBMI has the lowest (5.0k). The UEBMI and URBMI schemes have comparable predicted reimbursement rates (72.9% and 72.2%, respectively), which are much higher than that of the NCMS (53.3%). For outpatient treatments, the UEBMI scheme has the highest predicted gross cost (median 3.57k), followed by the URBMI (3.41k) and the NCMS (1.88k). The URBMI and UEBMI schemes have higher OOP costs (1.32k and 1.31k, respectively) than the NCMS (0.98k). Again, it is observed that the UEBMI and URBMI schemes have comparable predicted reimbursement rates (71.5% and 70.3%, respectively), which is higher than the NCMS (56.9%).

**Table 3:** Observed and predicted gross cost, OOP cost, and reimbursement rate.

			Gross cost	OOP cost	Reimbursement rate
**Inpatient**	**Observed**	UEBMI	14.22(5.21)	4.72(1.8)	0.725(0.03)
		URBMI	20.89(10.8)	8.18(3.88)	0.715(0.07)
		NCMS	14.55(5.27)	8.66(3.9)	0.525(0.05)
	**Predicted**	UEBMI	14.26(5.88)	5(2.27)	0.729(0.04)
		URBMI	19.9(11.8)	7.87(4.87)	0.722(0.09)
		NCMS	15.07(6.19)	8.47(4.08)	0.533(0.08)
**Outpatient**	**Observed**	UEBMI	3.28(1.01)	1.25(0.5)	0.711(0.06)
		URBMI	3.31(1.96)	1.21(0.65)	0.704(0.08)
		NCMS	1.68(0.81)	0.82(0.38)	0.56(0.11)
	**Predicted**	UEBMI	3.57(1.24)	1.31(0.55)	0.715(0.07)
		URBMI	3.41(2.02)	1.32(0.9)	0.703(0.12)
		NCMS	1.88(1.12)	0.98(0.57)	0.569(0.14)

In each cell, median (MAD). Gross and OOP cost: 1k RMB.

## Discussion

### Main findings

With historical reasons, multiple basic health insurance schemes have been co-existing in China, which has led to disparity between regions/populations. To reduce disparity and improve efficiency, consolidation is inevitable [21]. The consolidation process is challenging, with the huge amount of population, restricted resources, and complexity of the existing system. For all shareholders–individuals and governments, it is of significant interest to evaluate the differences across schemes after consolidation. Suzhou is one of the cities first selected for the pilot experiment, and what is learned from Suzhou can be informative for the regions that are undergoing or will undergo the consolidation.

In the literature, multiple studies have already examined the differences across insurance schemes [4–6]. However, they are all based on data collected prior to the consolidation, and very little is known about the existence and extent of differences after the consolidation. This study fills this knowledge gap. Different from those that investigated the design, execution, and management of the consolidation, this study analyzed patient-level data collected using a survey of random samples and is able to examine medical costs and insurance reimbursement from the patients’ (as opposed to the health care and insurance providers’) perspective.

Subjects’ characteristics were found to differ across insurance schemes in multiple aspects. NCMS covers rural areas, and UEBMI and URBMI cover different urban populations. The observed differences in education, occupation, and income are mostly attributable to the differences in the covered populations and have also been observed in published studies [1, 2, 19, 22]. A difference was also observed in health care accessibility, as measured using grade III hospitals. Under a fair health care system, patients should have equal access to health care facilities. The uneven distribution of health care resources has been observed in many early studies. Although having the consolidation and re-distribution of health care resources in the past a few years, it is unfortunate to observe that differences in health care accessibility still exist. Grade III hospitals and high-quality health care services are mostly located in cities, especially large cities, making subjects in the rural areas covered by NCMS disadvantaged. More effort is needed on redistributing health care resources and making high-quality resources available to people in the rural areas. For outpatient treatments and those who used insurance, the UEBMI scheme has a higher number of treatments and, accordingly, higher treatment costs. In the survey, no significant difference with regard to demographics (gender and marital status) was observed. A limitation of this study is that, without having access to medical record data, health conditions of the subjects are unknown. However, there is no indication, based on this study and those published, that subjects covered by UEBMI were less healthy. The higher number of outpatient treatments can be caused by the easier access to health care and other factors.

The most important finding is that, after effectively eliminating the differences in subjects’ characteristics, the three insurance schemes still differ significantly in terms of gross and OOP costs and insurance reimbursement rates. For inpatient treatments, the difference between the median gross cost under UEBMI and URBMI is as high as 5.64k. Analyzing the OOP cost, which more directly measures the financial burden on patients, shows that those under NCMS have cost 0.6k and 3.47k higher than those under URBMI and UEBMI, respectively. Subjects covered by NCMS live in rural areas and tend to have lower incomes. The higher OOP cost and lower economic status make them especially disadvantaged. Those covered by NCMS also have a median predicted reimbursement rate lower by almost 20% than those covered by UEBMI or URBMI. In principle, those under the three different schemes have the same access to health care resources and utilization of insurance. The differences observed in this study can be attributable to multiple factors. For example, subjects living in the rural areas and covered by NCMS tend to be less knowledgeable of the medical and insurance systems and hence do not use the system as effectively. Another possibility is that the consolidation may be not as effective as originally expected. More policy and management developments are needed to make the three schemes “more similar”. Outpatient treatment usually corresponds to less severe illness conditions, and so the cost per episode is much lower. However, the difference in cost still persists. For outpatient treatment, the insurance utilization rate is less satisfactory. Fig 1 suggests that coverage depth needs to be improved, as the leading cause of not using insurance is “treatment not covered”. Other effort may also be needed to improve the effectiveness of insurance for outpatient treatment. The low reimbursement rate under NCMS is similar to that for inpatient treatment and demands similar attention. With the significant differences between inpatient and outpatient treatments, independent effort may be needed to eliminate the difference in outpatient reimbursement rate.

Compared to other areas in China, Suzhou started the consolidation relatively earlier. In addition, Suzhou has a higher economic status. Especially, its rural areas are considerably richer than other rural areas in China. A higher economic status and hence less financial constraints usually correspond to better health care and health insurance conditions. As such, it is reasonable to expect similar or more severe across-scheme differences in other parts of China.

### Limitations

This study has limitations. With limited resources, the sample size is limited, and all the data were collected in Suzhou. As in other survey studies, there is a risk of selection bias and recall bias. Every possible effort has been made in the study design and sampling to ensure the representativeness of the samples. Statistical analysis does not suggest any obvious selection bias or recall bias. Health condition can be an important factor for the proposed analysis, and this study is limited in collecting limited information on subjects’ health condition. Using information on inpatient treatment days, number of outpatient visits, and utilization of different hospitals may alleviate this problem to a large extent. As the consolidation is still being experimented within selected cities and provinces, our study is limited to specific regions. The results in this study may not be fully generalizable to the whole country. Another limitation is that the collected information is not sufficient to identify the causes of the observed differences. More detailed information collection will be needed.

### Conclusion

This study has provided concrete evidences that, after the consolidation, there are still significant differences in medical costs and reimbursement rates across the three basic insurance schemes in China. Such differences exist independent of the differences in subjects’ characteristics. More research is needed to fully understand the causes of such differences, and tailored interventions are needed to eliminate them.

## Abbreviations

UEBMI: Urban Employee Basic Medical Insurance
URBMI: Urban Resident Basic Medical Insurance
NCMS: New Cooperative Medical Scheme
OOP: out-of-pocket
GDP: gross domestic product
MAD: median absolute deviation
